# From Clinician to Suspect Case: My Experience After a Needle Stick in an Ebola Treatment Unit in Sierra Leone

**DOI:** 10.4269/ajtmh.14-0769

**Published:** 2015-02-04

**Authors:** Lewis Rubinson

**Affiliations:** R. Adams Cowley Shock Trauma Center, University of Maryland School of Medicine, Baltimore, Maryland

While providing clinical care in the confirmed ward of the Ebola Treatment Unit (ETU) at the Kenema Government Hospital (KGH) in Kenema, Sierra Leone, I accidentally stuck an 18-gauge hollow-bore needle deep into my left thumb. I could immediately feel some blood oozing under my gloves, and I squeezed the area of penetration to try to promote additional bleeding. I rinsed the outside of my gloves with the only available option—0.5% bleach. In an ETU, one cannot simply remove one's gloves and clean one's hands with soap and water as one would with a needle stick in other clinical environments. After the momentary shock and embarrassment subsided, I notified my clinical partner about what transpired and then called by radio to have an urgent egress from the ETU.

When I arrived in the personal protective equipment (PPE) doffing area, Dario Gramuglia, chief of logistics for the World Health Organization (WHO) at Kenema, maintained full discipline, and we adhered to the stringent protocol of proper PPE removal without deviation; in all honesty, I was quite anxious to examine the needle stick and clean it properly as soon as possible. When my inner glove was removed, I had blood on my thumb and thenar eminence. I now was able to confirm that it was a fairly deep penetration. I first cleaned the wound with water and had trouble finding soap, because 0.05% bleach was frequently used for handwashing. Ultimately, I had to make do with 2% chlorhexidine gluconate swabs to clean the wound.

As I walked up the hill from the doffing station toward the WHO office, I now had time to begin contemplating what my risks were of exposure to Ebola virus disease (EVD) and other blood-borne pathogens. The needle had been stuck in the side of a plastic intravenous (IV) bottle, a practice some local nursing staff used to extract crystalloid fluid into syringes to create flushes. Usually, clean needles were used for such practices, but the IV for this particular patient had been started at a different isolation unit before her arrival in Kenema; therefore, the needle's history was unknown, meaning it was uncertain whether the needle had been used to draw blood from any patient. Still, as I considered the risk from the needle itself then and over the following days, I always considered it to be fairly low. The real risk in my mind, which was confirmed by other consultants, was whether the needle carried infectious materials from the outside of my glove into my thumb. Before the needle stick, I had been assisting a confused person with EVD to return to the confirmed ward, and then shortly afterward, I was examining and providing parenteral crystalloid therapy to several severely ill persons with EVD. My gloves did not have visible blood on the outside before the needle stick, because if so, I would have disinfected with 0.5% bleach whenever such a circumstance was noted. Had there been blood on my glove, I would have been fairly concerned, but I also would have been partially reassured; in a past investigation, blood on a healthcare worker's glove only yielded Ebola RNA, and infectious virus could not be cultured.^1^ Still, those data were limited. This event would be considered a high-risk exposure.^2^ The public health definition of high-risk exposure does not necessarily equate with high probability of sequelae. As I digested the event, I believed that my risk of becoming ill with EVD was low but not zero. Nevertheless, it was much more likely I would never get ill. Because I met the definition for a high-risk exposure, I was to be medically evacuated from Sierra Leone back to the United States.

Hundreds of healthcare workers have become ill with EVD during the 2014 West African outbreak.^3^ Most of these people did not realize that they were exposed until they became ill. I found myself in a different situation. Statistically speaking, it was unlikely that I would become ill with EVD, but I had a specific exposure, which increased my risk above the everyday risk of working in the ETU. I now just had to wait for the future to find out what would be the consequence of the needle stick. It was also very hard for me to reconcile that I was not ill and likely never to be ill, but I was to be assisted with tremendous resources to get me back home. I am extremely grateful for all of those who made that happen. At the same time, many West Africans were actually ill or dying with EVD, and most were unable to get anything beyond very basic healthcare. This truth would continue to plague me throughout my experience.

We frequently witnessed as many as half a dozen or more persons with EVD at a time transported for hours in makeshift ambulances in sweltering heat without water and brought to KGH, a referral treatment unit. When they arrived dehydrated and prostrate, even the most basic acute care functions, such as provision of adequate levels of hydration and use of clinical laboratories to guide rehydration, remained elusive. Many died on the floor or soiled mattresses without access to basic comforts, such as pillows or blankets. Many died alone. Together with our Sierra Leonean colleagues, we tried system fixes, but they consistently seemed to be “a bridge too far”.

I had to hastily leave KGH to get to past the EVD control checkpoints and be assured that I would be in Freetown when the aeromedical evacuation plane arrived. In retrospect, this was one of the hardest emotional burdens from the experience. There was no planned transition from the intense work and bearing witness of tragedy in Kenema to all of a sudden being en route back to the United States. My colleagues and I had been working to support the changeover of the KGH to a small isolation unit, and this switch had been nearly completed, but not fully, at the time of my needle stick. I left believing I had not successfully completed the mission.

At the time of my needle stick, there were two post-exposure prophylaxis (PEP) options: a live attenuated virus vaccine or a small interfering RNA therapeutic.^4^ Experience in humans for both product was extremely limited. I figured that my risk of EVD was low, but I wanted to make it as close to zero as possible. It is also worth noting the context of my decision. At the time, only three persons with EVD had been treated in the United States, and one had been critically ill. Several healthcare workers who had been evacuated to Europe had died. Although many of us at the time believed that the mortality rate with early full-resource care, such as is available in specialized centers in the United States, would be considerably lower, in September of 2014, that still was yet to be shown. Hence, if I did get ill, there was no telling how I would fare. I, therefore, chose to enroll in one of the PEP studies. I received an investigational PEP intervention flown from a study site in the United States within minutes of stepping on the plane that would fly me back to the United States; amazingly, this was only 43 hours after my exposure.

En route to the United States was the first time that I had insight into the fear and politics of EVD that would engulf the United States during the next weeks. While refueling in the Azores, the US Air Force leadership on site made it very clear that we were not to open the door to the Gulfstream. I was approximately 50 hours from exposure and asymptomatic: not a risk to anyone, but someone who was feared. We then landed at a small airport in Maryland, and a news crew was trying to get footage. There, of course, was no story to tell. The real story was the tragedy unfolding in West Africa.

I was transported to the Biocontainment Unit of the National Institutes of Health (NIH). When I arrived, I was febrile, with escalating rigors and chills. A terrible headache with photophobia soon ensued, and I had significant myalgia. I was fairly sure that my symptoms were consistent with the PEP intervention; however, my anxiety started to increase as I lay in bed freezing under a multitude of blankets. After caring for hundreds of persons with EVD, I knew that my symptoms were consistent with early EVD. I reassured myself that my condition was more likely to be explained by the PEP. This belief was reinforced by my conversations with international experts who happen to be friends as well.

The staff at the NIH was well-trained, very caring, and professional. Still, I was going through this alone. My 6-year-old daughter was still under the impression that I was in Africa. I held the purpose of my trip to Africa from her, because how do you explain EVD to a child without generating enormous anxiety in her? Now, I was back in the United States and at risk. I missed her tremendously, but no one could visit me, and of course, I did not want to put her at risk either physically or emotionally. Also, if my name got released and with the fear and lack of scientific-derived actions in the United States, I was worried she would be shunned by some people who have children at her school. I, therefore, kept my isolation and illness a secret from many friends and colleagues. This was an important decision, but at the same time, it left me isolated from many who I care about.

Severe nausea and copious diarrhea fortunately never occurred, and several days after arriving at the NIH, my early symptoms improved. It became increasingly clear that my initial symptoms were a response to the PEP and did not represent early EVD. I felt relieved. All I wanted to do now was go home. Instead, I remained in quarantine at the NIH for multiple days. When I transitioned to home quarantine, science clearly was not informing the restrictions to which I had to adhere. I was fully asymptomatic, but I was not allowed to leave my house at all, including not being able to walk my dog off my property. I, of course, never would want to harm anyone, but the restrictions seemed out of proportion to any risk. I realize the public health agency did not want to have to investigate possible contacts if I did become ill with EVD. It is worth noting that the contact-tracing investigations from the three cases in Dallas as well as the one case in New York City have never identified a subsequent case. Public health agencies are supposed to be the scientific defenders to ensure that politics do not cause knee-jerk reactions to outbreaks. In honesty, these organizations are led by political appointees, and ultimately, science has, on occasion, been overrun by fear and political posturing.

When my symptoms abated, I retransitioned back to clinician from suspect case. I have engaged in escalating preparations for sporadic cases in the United States, activities that have been fraught with many challenges. Outside of the specialized biocontainment units, US hospitals are largely inexperienced with EVD, and many preparedness decisions have been based on fear or dogma rather than scientific truth or common sense. I hope that we start getting it right so that there will be no additional clinicians in the United States who have to face being suspect or confirmed cases.

Was the aeromedical evacuation, PEP, and overall resource expenditure overkill for the risk? I remain ambivalent. The risk was real, but it was far from a foregone conclusion that I would develop EVD. If the healthcare infrastructure was more functional in Sierra Leone, I could have undergone evaluation and possible PEP in that country. I am glad that tremendous resources are available for persons who may contract EVD. I just wish that they were available to all and not just the handful of expatriate responders.
